# Degree of coronal alignment correction can’t predict knee function in total knee replacement

**DOI:** 10.1186/s12893-021-01372-3

**Published:** 2021-10-30

**Authors:** Shibai Zhu, Xiaotian Zhang, Xi Chen, Yiou Wang, Shanni Li, Wenwei Qian, Huiming Peng, Wei Wang, Jin Lin, Jin Jin, Xisheng Weng

**Affiliations:** 1 Department of Orthopedics, Peking Union Medical College Hospital, Peking Union Medical College and Chinese Academy of Medical Sciences, Beijing, 100730 China; 2 Department of General Surgery, Peking Union Medical College Hospital, Peking Union Medical College and Chinese Academy of Medical Sciences, Beijing, 100730 China; 3 Department of Orthopedics, Beijing Tongren Hospital, Capital Medical University, Beijing, 100730 China

**Keywords:** Total knee replacement, Neutral alignment, Correction rate, Hospital for special surgery knee score

## Abstract

**Background:**

Whether neutral alignment brings better clinical outcomes is controversial. Consideration of the preoperative knee condition of patients and some limitations of previous studies, we suggested that other index may be more important than a generic target of 0° ± 3° of a neutral axis to reflect changes in coronal alignment after total knee replacement (TKR). The purpose of this study was to explore the relationship between alignment and functional outcome with a new grouping method and the concept of correction rate.

**Methods:**

The study included 358 knees, the mean follow-up period was 3.62 years. A new grouping method was adopted to divide patients into three groups based on the degree of correction of mechanical femoral—tibial angle (MFTA): under-correction (n = 128), neutral (n = 209) and over-correction (n = 21). Hospital for Special Surgery (HSS) score were compared among the 3 groups (ANOVA with or without LSD t-test). In addition, we also attempt to further explore whether the concept of correction rate can predict postoperative functional score (Simple linear correlation analysis).

**Results:**

HSS score showed significant improvement in all groups. There was no difference in HSS score (88.27 vs 88 vs 85.62) (*p* = *0.88*) or incremental scores (26.23 vs 25.22 vs 22.88) (*p* = *0.25*) based on the postoperative alignment category for the degree of correction of MFTA at the last follow-up. The correlational analyses also didn’t show any positive results (*r* = *-0.01 p* = *0.95, r* = *-0.01 p* = *0.97, r* = *0.11 p* = *0.15, r* = *0.01 p* = *0.90*).

**Conclusion:**

Categorization of optimal coronal alignment after TKR may be impractical. But we still believe that the concept of correction rate and new grouping method are worthy of research which can reflects the preoperative knee condition and the change of coronal alignment. Perhaps it can be better used in TKR in the future.

*Level of evidence*: III.

## Introduction

The traditional opinion is that the postoperative limb alignment should be neutral, which is considered the prerequisite for successful total knee replacement (TKR) [1–3]. However, recent studies have challenged the gold standard of neutral alignment, questioning the utility of defining alignment as a dichotomous variable (aligned or malaligned) [4, 5]. With the controversial discussion on dissatisfaction following TKR [6–9], dynamic loads [10], constitutional [11] or kinematical alignment [12], many surgeons do not accept the neutral mechanical alignment as the golden standard anymore.

With the improvement of modern implants and fixation techniques, many studies have confirmed the coronal alignment may not be as important a cause of failure as has been previously thought [4, 5]. However, the relationship between alignment and knee function remains elusive (Table 1) [13–25]. Some research showed that residual varus leads to better [14, 21] or similar [13, 17–19, 23] clinical outcome for preoperative varus osteoarthritis. Recently, Zhang et al. demonstrated slight under-correction for a valgus knee results in same knee function score compared with neutral alignment [24].

**Table 1:** Research on the relationship between coronal alignment and clinical outcomes in recent 10 years (Excluding navigation or robot-assisted technologies)

Study	Follow-up	Preoperative alignment	Grouping method based on postoperative alignment				Results or conclusion
Magnussen et al. [13]	5.8y	Varus	Neutral (0 ± 3°), n = 352	Varus (< − 3°), n = 181	–	–	No difference in IKS
Magnussen et al. [13]	5.8y	Severe varus (≤ − 10°)	Neutral (0 ± 3°), n = 131	Varus (< − 3°), n = 131	–	–	Varus was better in IKS
Vanlommel et al. [14]	7.2y	Varus	Neutral (0 ± 3°), n = 75	Mild varus (-3° to -6°), n = 46	Severe varus (< − 6°), n = 22	–	Mild varus was better in KSS and WOMAC
Manjunath et al. [15]	3y	All	Neutral (0 ± 3°), n = 96	Outlier (< − 3° or > 3°), n = 24	–	–	Neutral was better in KSKS, but but no difference in KSFS
Stucinskas et al. [16]	1y	All	Neutral (0 ± 3°), n = 62	Outlier (< − 3° or > 3°), n = 29	–	–	No difference in KSS
Hatayama et al. [17]	6.1y	Varus	Neutral (0 ± 3°), n = 70	Varus (≤ − 3°), n = 47	–	–	No difference in HSS
Nishida et al. [18]	3.6y	Varus	Neutral (0 ± 3°), n = 128	Mild varus (− 3° to − 6°), n = 61	Severe varus (< − 6°), n = 15	Valgus (> 3°), n = 16	Neutral and mild varus was better in KSFS, but no difference in KSKS
Rames et al. [19]	1.3y	Varus	Neutral (0 ± 3°), n = 149	Mild varus (-3° to -6°), n = 60	Severe varus (< -6°), n = 28	Valgus (> 3°), n = 19	No difference in OKS, FJS and SF-12
Dominique et al. [20]	8.5y	Severe varus (≤ − 10°)	Group 1 (< 0°), n = 88	Group 2 (≥ 0°), n = 62	–	–	No difference in IKS and OKS
Schiffner et al. [21]	3–5y	Varus	Neutral (0 ± 3°), n = 115	Varus (≤ -3°), n = 33	–	–	Varus was better in KOOS, but no difference in OKS
Abdel et al. [22]	20y	All	Neutral (0 ± 3°), n = 69	Outlier (< − 3° or > 3°), n = 12	–	–	No difference in KSS
Lee et al. [23]	5y	Valgus	Neutral (0 ± 3°), n = 69	Mild valgus (3° to 6°), n = 17	Severe valgus (> 6°), n = 7	–	No difference in KSS and WOMAC
Zhang et al. [24]	5.2y	Varus	Neutral (0° to − 3°), n = 86	Mild varus (− 3° to − 6°), n = 62	Severe varus (< − 6°), n = 27	Valgus (> 0°), n = 44	Neutral and mild varus were better in KSS and WOMAC compared with severe varus and valgus
Bilgin et al. [25]	7.08y	All	Neutral (0 ± 3°), n = 20	Outlier 1 (− 3° to − 6° or 3° to 6°), n = 24	Outlier 2 (< − 6° or > 6°), n = 26	–	Neutral and outlier 1 were better in OKS and SF-36 compared with outliers 2

*HSS* Hospital for Special Surgery knee score, *IKS* International Knee Society score, *OKS* Oxford Knee Score, *KSS* Knee Society Score, *KSKS* Knee Society Knee Score, *KSFS* Knee Society Functional Score, *WOMAC*  Western Ontario and McMaster Universities Arthritis Index, *FJS* Forgotten Joint Score, *KOOS* Knee Injury Osteoarthritis Outcome Score

All studies aimed to obtain a neutral postoperative mechanical axis and used conventional procedure to perform TKR. Patients in all of the above studies underwent full-length hip-knee-ankle radiographs to evaluate the pre and postoperative mechanical femoral-tibial angle

Based on the inconsistency of these findings and some limitations of the above studies (see discussion for limitations), a new method was adopt to explore the relationship between alignment and clinical outcomes, that is, grouping the patients according to the degree of correction of the limb alignment (under-correction, neutral and over-correction). In addition, we also introduce a new, more intuitive and accurate index (the concept of correction rate) for the severe deformity to analyze whether there is a correlation between it and clinical outcomes.

The hypothesis of our research is: (1) Patients in the under-correction and neutral alignment groups received a better clinical outcome than those in the over-correction group; (2) Postoperative knee function score can be predicted by the value of correction rate. (that is to say, when a certain value is reached, the knee function score is the highest, lower or higher than this value, and the score gradually decreases).

## Patients and methods

### Patients

**Fig. 1:**
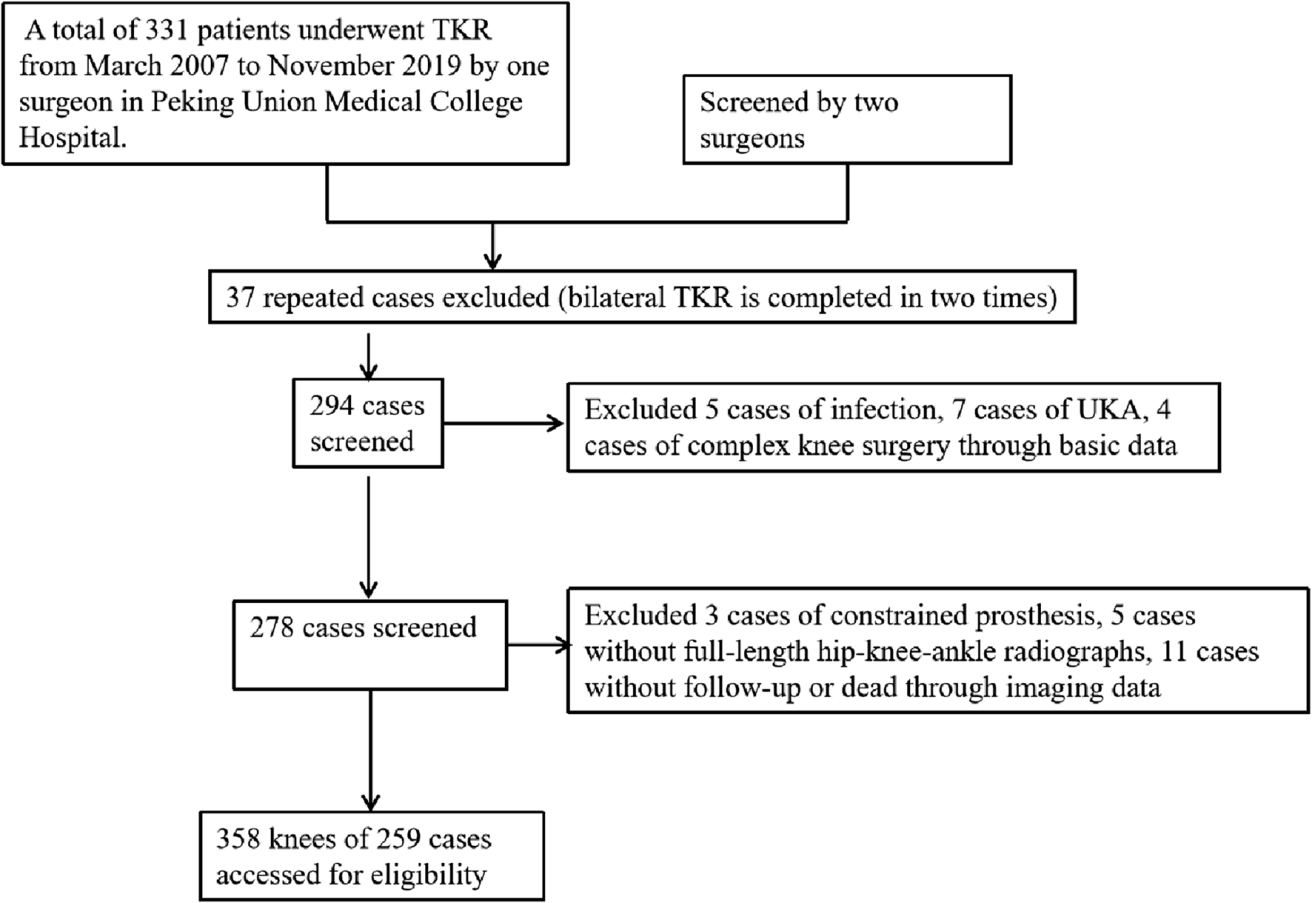
Flow diagram of study

A retrospective analysis was conducted on all TKR patients by one doctor at our Peking Union Medical College Hospital between March 2007 and November 2019 (approval number: S-K778). The inclusion criteria were patients with a history of osteoarthritis or rheumatoid arthritis that received a primary TKR, and used the modern unconstrained prosthesis and the cemented fixation technique. Our aim was to explore the effect of the degree of correction on the postoperative HHS score, so we did not exclude the knee with severe deformity. Besides, the preoperative and postoperative weight-bearing full-leg radiographs must be obtained for all included patients. After screening, excluded cases were as follows: 5 cases of infection, 7 cases of unicompartmental knee replacement, 4 cases of complex knee surgery, 5 cases without weight-bearing full-leg radiographs, 3 cases of constrained prosthesis, 11 cases without follow-up or death (Fig. 1). Finally, 259 patients (358 knees) met the inclusion criteria were included in our study. The demographic data of all patients are shown in Table 2.

**Table 2:** Demographic data

Patients	259
Knees	358
Side (Left/Right/Bilateral)	83/77/99
Etiology (OA/RA/Other)	330/28
Prosthesis type (CR/PS)	181/177
Age (year) (Mean ± SD, range)	66.21 ± 7.72 (28 ~ 86)
Gender (Male/Female)	49/210
BMI (Mean ± SD, range)	26.39 ± 3.81 (17.38 ~ 37.19)
Follow-up time (year) (Mean ± SD, range)	3.62 ± 2.61 years (0.42 ~ 13.08 years)

All TKR were designed to obtain the postoperative coronal mechanical neutral alignment and performed by one surgeon. The distal femur and proximal tibia were resected perpendicular to the mechanical axis using an intramedullary and extramedullary alignment guide, respectively. The same rehabilitation program was adopted for all patients. And the pre and postoperative (2–4 weeks after surgery) weight-bearing full-length hip-knee-ankle radiographs were taken. Preoperative Hospital for Special Surgery (HSS) score was evaluated on all patients and the HSS score at the last follow-up were used as the postoperative score. All the assessment including radiographic measurement and HSS score were performed by two independent observers (Zhu and Chen, resident with 3 years of experience in joint surgery) according to a standardized protocol.

### Grouping criteria

**Fig. 2:**
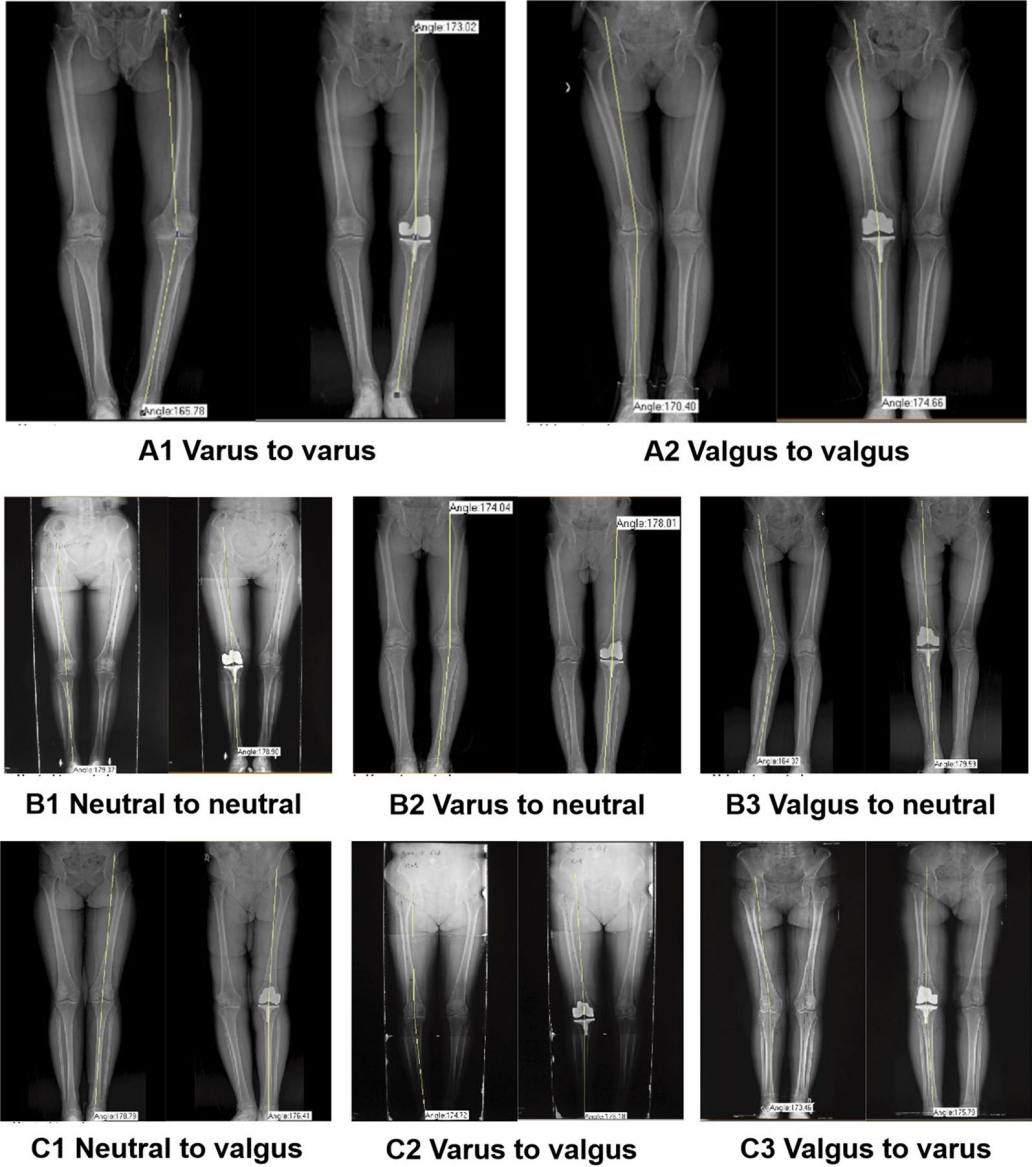
**A** Diagram of under-correction group (varus to varus, valgus to valgus). **B** Diagram of neutral alignment group (neutral to neutal, varus to neutral and valgue to neutral). **C** Diagram of over-correction group (neutral to valgus, varus to valgus and valgus to varus)

Patients were divided into 3 groups according to the degree of correction of limb alignment, namely under-correction group (A), neutral alignment group (B), and over-correction group (C). In group A (Fig. 2A), the postoperative mechanical femoral-tibial angle (MFTA) was beyond 0° ± 3°, but no excessive correction. In group B (Fig. 2B), the postoperative MFTA was within 0° ± 3°. In group C (Fig. 2C), the postoperative MFTA was beyond 0° ± 3°, and this group of patients specifically refers to which preoperative varus (valgus) knee was corrected to valgus (varus) knee, and the preoperative neutral alignment knee was corrected to the valgus or varus knee.

### Special instructions

In this study, we first introduced the concept of correction degree and correction rate. The varus knee was set to a negative angle and the valgus knee was set to a positive angle. The neutral position refers to the MFTA within 0° ± 3°. The correction degree refers to MFTA (preoperative)—MFTA (postoperative) or MFTA (postoperative) – MFTA (preoperative). The correction rate refers to correction degree/MFTA (preoperative). The correction degree and correction rate can be negative.

Examples of varus knee:

If the preoperative MFTA was varus of 6° and the postoperative MFTA was varus of 2°, then the correction degree was 4° and the correction rate was 66.7%. (Fig. 2B2).

If the preoperative MFTA was varus of 6° and the postoperative MFTA was varus of 8°, then the correction degree was − 2° and the correction rate was − 33.3%.

If the preoperative MFTA was varus of 6° and the postoperative MFTA was valgus of 4°, then the correction degree was 10° and the correction rate was 166.7%. (Fig. 2C2)

During the research, we found that the correction degree and correction rate were zero or infinity for the knee with neutral alignment or mild deformity, which is not in accordance with the actual situation. So we only performed the correlation analysis in preoperative severe deformity (varus or valgus) more than 10°.

According to our research purposes, HSS score, incremental scores of HSS were recorded to compare whether there was statistical difference among the three groups. In addition, a scatter plot of MFTA and HSS score was drawn and analyzed to verify whether neutral alignment represented better knee function. Finally, the correlation analysis between TKR correction rate, correction degree, and HSS score and incremental scores of HSS was studied by relevant statistical methods in the patients with severe deformity to explore further the relativity between correction rate and HSS score.

### Statistics analysis

All data were expressed as the mean standard deviation and analyzed with Statistical Package for the Social Sciences(SPSS) version 22.0 (Chicago, IL). Differences categorical data were compared using the chi-square tests and Fisher-Freeman-Halton test. All continuous variables were evaluated using the ANOVA with or without least significant difference (LSD) t-test when there was a normal distribution, and non-normal distributions were compared using the Kruskal–Wallis test. In all comparisons, a p value < 0.05 was considered significant. Simple linear correlation analysis wiht 95% confidence intervals was used to explore the relationship between correction rate and knee function score.

## Results

### Comparison among groups

#### Demographic data

The basic data of the three groups of patients is shown in Table 3, including 128 knees in group A (25 males and 103 females), 209 knees in group B (39 males and 170 females), and 21 knees in group C (three males and 18 females). Two patients in group B with patellofemoral problems underwent revision surgery 1 year and 4.4 years postoperatively, respectively. There was no statistical difference in gender (*p* = *0.85*), age (*p* = *0.35*), follow-up time (*p* = *0.84*) or preoperative HSS score (*p* = *0.88*) among the three groups. However, for the BMI, there was a statistical difference between group A and group B (*p* = *0.01*). Moreover, the preoperative MFTA in group A is greater than group B and C with statistically significant (*p* < *0.01*). And the radiographic measurements of postoperative MFTA was different among the three cohorts as expected *(p* < *0.01)*.

**Table 3:** Basic data of three groups

Variable	A (*n* = 128)	B (*n* = 209)	C (*n* = 21)	*P value*
Gender (M/F)	25/103	39/170	3/18	^a^0.849
Age (years)	65.89 ± 7.48	65.58 ± 8.35	63.19 ± 6.15	^b^0.35
BMI (kg/m^2^)	27.35 ± 4.19	26.13 ± 3.66^*^	27.41 ± 3.54	^**bc**^**0.01**
Follow-up (year)	3.62 ± 2.72	3.49 ± 2.41	3.74 ± 1.96	^b^0.8386
Preoperative HSS score	62.05 ± 12.81	62.78 ± 12.84	62.74 ± 12.19	^b^0.876
Preoperative MFTA	− 11.19 ± 6.25	− 6.05 ± 6.59^*^	− 3.58 ± 5.77^*^	^**bc**^**0.001**
Postoperative MFTA	− 4.66 ± 2.53	− 0.65 ± 1.51	3.06 ± 3.16	^**b**^** < 0.0001**

Bold indicates statistically significant

The values are given as the mean and the standard deviation; *MFTA* mechanical femoral-tibial angle, *HSS score* Hospital for Special Surgery score

*A* Under-correction group, *B* Neutral alignment group, *C* Over-correction group

^a^chi-square test; ^b^ANOVA; ^c^LSD-t test; ^*^There was a statistically difference compared with the A group, however, no statistically difference in other two groups

#### MFTA and HSS score

The HSS score and MFTA for the entire cohort show in Table 4. HSS improved significantly from a preoperative mean of 62.51 ± 12.76 to a postoperative mean of 87.96 ± 6.80 at the last follow-up (*P* < *0.01*). MFTA was corrected from a preoperative mean of -7.74 ± 6.93 to a neutral level of -1.86 ± 3.04 after surgery.

**Table 4:** Preoperative and postoperative MFTA, HSS score in 358 knees

Preoperative MFTA (358 knees, Mean ± SD, range)	− 7.74 ± 6.93 (− 27 ~ 15.53)
Postoperative MFTA (358 knees, Mean ± SD, range)	− 1.86 ± 3.04 (− 13 ~ 7.38)
Preoperative HSS score (358 knees, Mean ± SD, range)	62.51 ± 12.76 (28 ~ 78)
Postoperative HSS score (358 knees, Mean ± SD, range)	87.96 ± 6.80 (41 ~ 99)

varus −, valgus + . MFTA = mechanical femoral-tibial angle

#### HSS scores

**Fig. 3:**
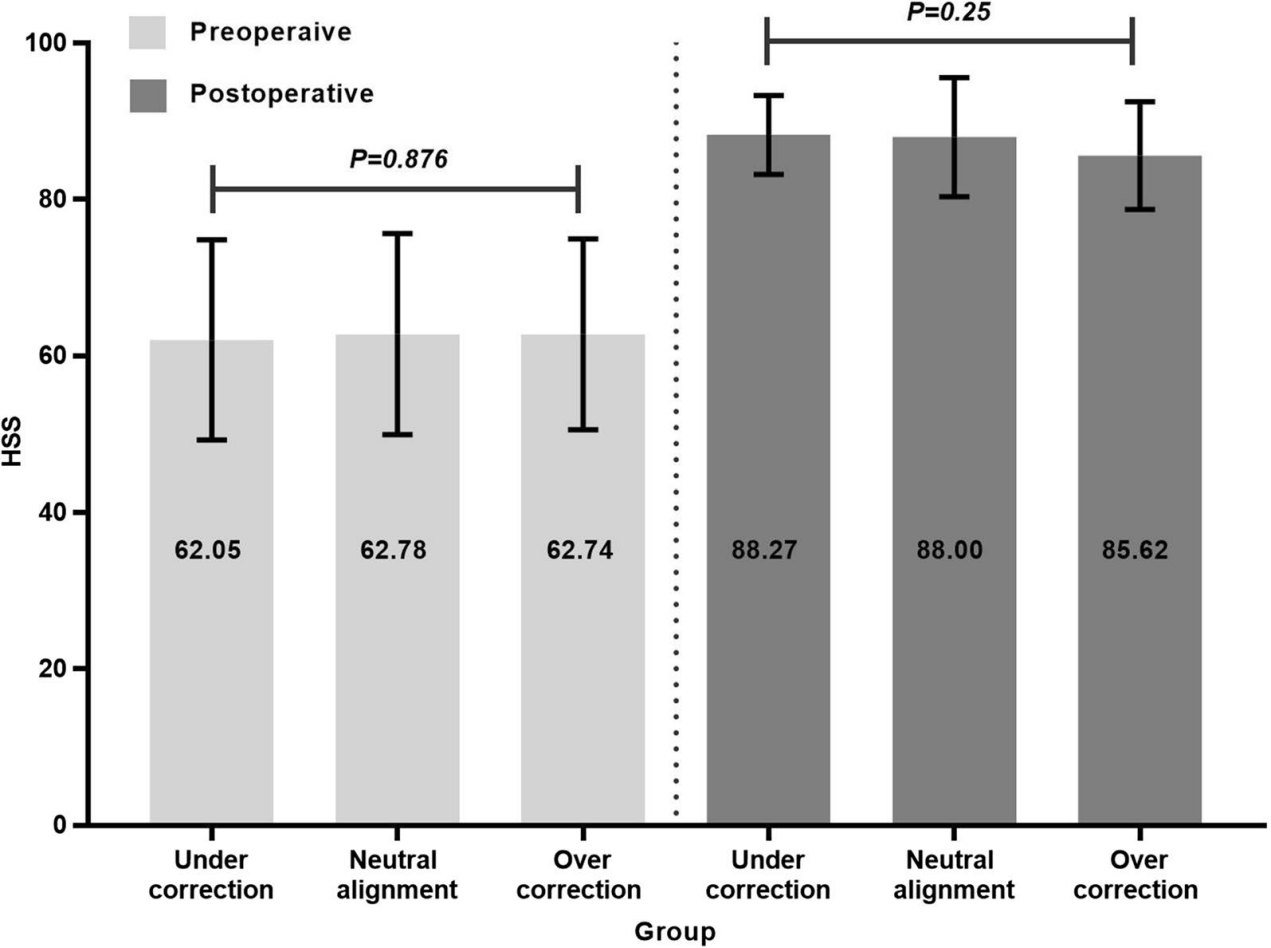
Comparison of pre and postoperative HSS score among three groups

**Fig. 4:**
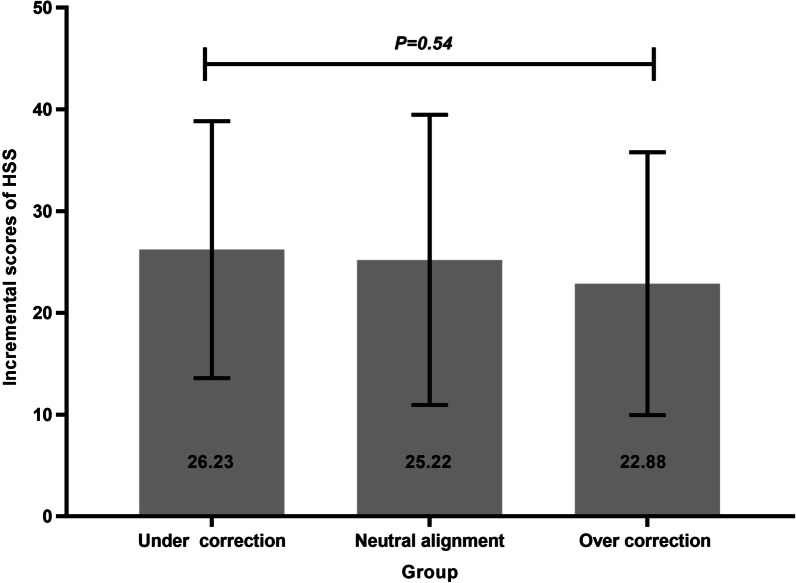
Comparison of incremental scores of HSS among three groups

For all study groups, HSS score improved following TKR. The postoperative HSS score of groups A, B, and C were 88.27 ± 5.06, 88.00 ± 7.61, and 85.62 ± 6.91. The incremental HSS score of groups A, B, and C were 26.23 ± 12.62, 25.22 ± 14.26, and 22.88 ± 12.92. There was no significant difference in HSS score (*p* = 0.25) or incremental scores (*p* = 0.54) among the three groups (Fig. 3, 4).

### Correlation analysis between postoperative MFTA and HSS score

**Fig. 5:**
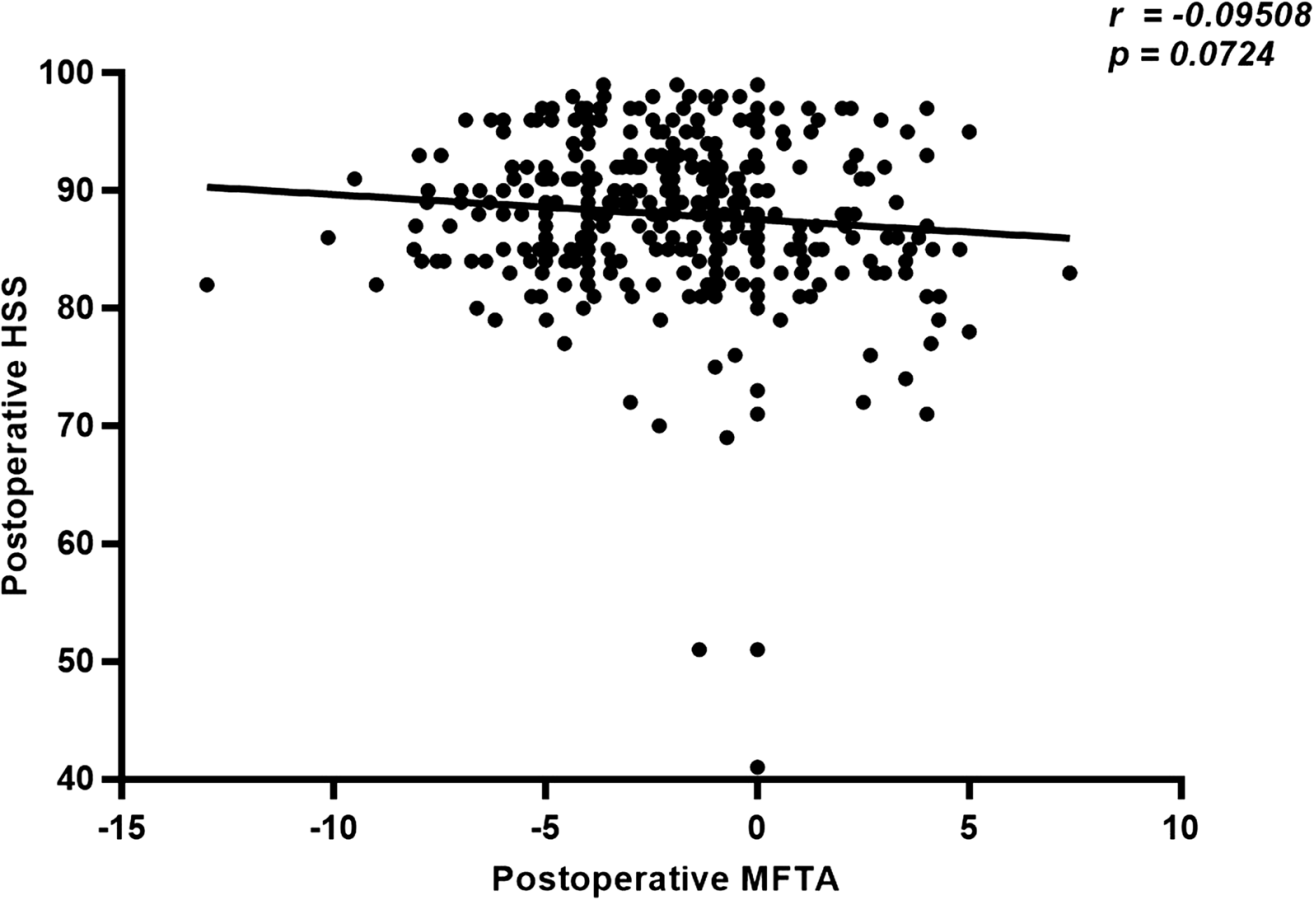
Correlation analysis between postoperative HSS score and MFTA

There was no correlation between postoperative MFTA and HSS score in all 358 knees (R = − 0.095, p = 0.072, CI: − 0.1968 to 0.008652) (Fig. 5).

### Correlation analysis between correction degree of alignment and HSS score in the patients with severe deformity

**Fig. 6:**
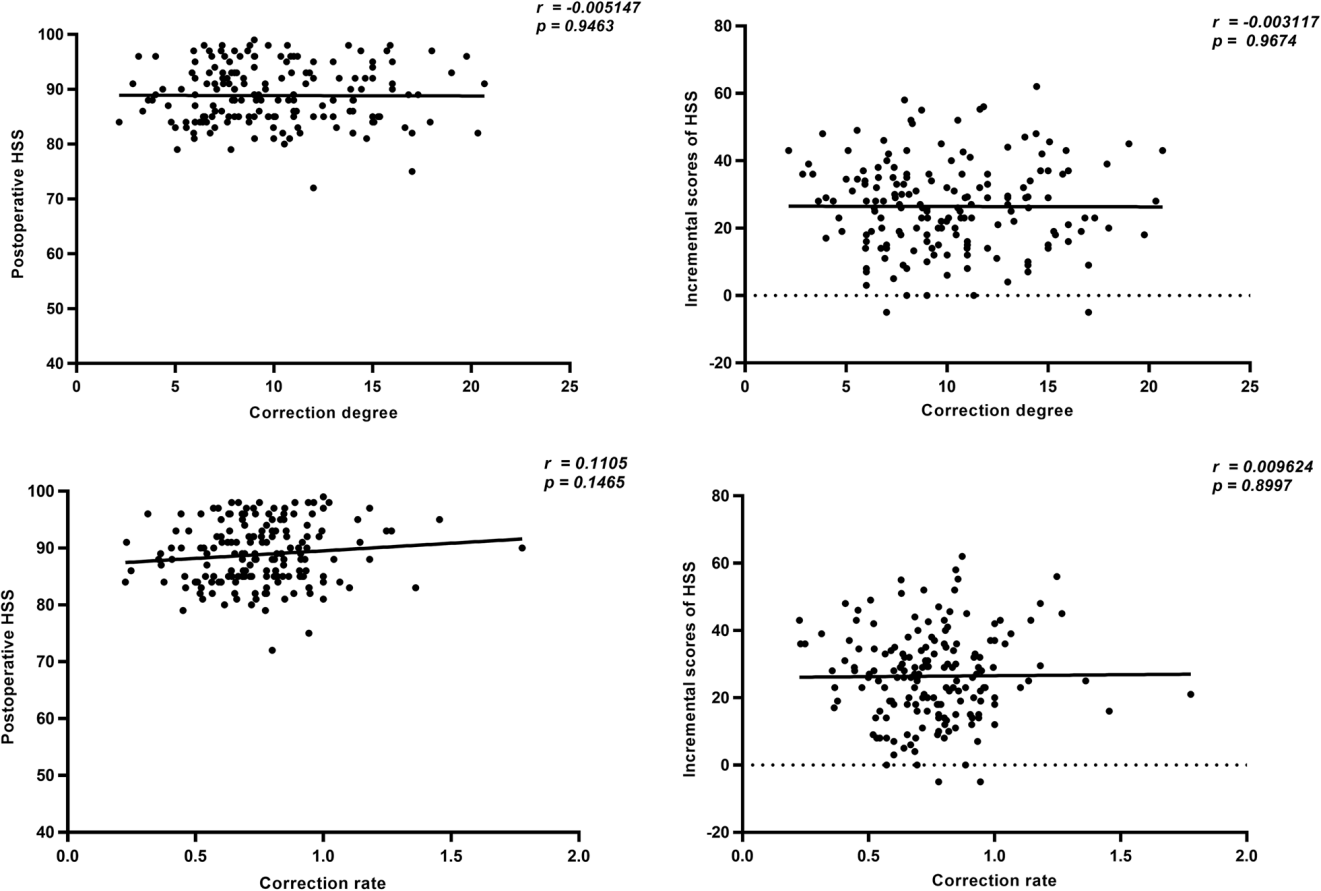
Correlation analysis in the knee with severe deformity

The severe deformity was set to the preoperative MFTA beyond 0° ± 10°. 174 knees meet the criteria (165 varus and 9 valgus), but we still did not find any correlation between correction degree and HSS score (R = − 0.01, p = 0.95, CI: − 0.15 to 0.14), correction degree and incremental scores of HSS (R = -0.01, p = 0.97, CI: -0.15 to 0.15), correction rate and HSS score (R = 0.11, p = 0.15, CI: − 0.04 to 0.26), or correction rate and incremental scores of HSS (R = 0.01, p = 0.90, CI: -0.14 to 0.16) (Fig. 6).

## Discussion

The principle of the mechanical alignment made the prosthesis placed in the neutral position always the gold standard of TKR surgery [2, 3]; this was considered to be closely related to the postoperative and the survival rate of the prosthesis. Despite the use of older designed prostheses and short-leg radiographs, the early study of the alignment after TKR confirmed the above mentioned view from clinical, imaging, simulator, cadaver, finite element, and retrieval research [26–32]. And even now, there are studies confirming the importance of neutral alignment for the long-term survival rate of prostheses [33]. However, since a study from Mayo Clinic pointed out that neutral alignment does not represent a better prosthesis survival rate through a 15 year and 20 year follow-up study [4, 22], the controversy about neutral alignment is also increasing, and many surgeons even do not accept the neutral mechanical alignment as the golden standard anymore.

In addition to the survival rate of prosthesis, many scholars paid attention to the relationship between coronal alignment and clinical outcomes (Table 1) [13–25]. But we think these studies have some limitations. For example, some studies [15, 16, 22, 25] did not take into account the preoperative varus or valgus of the knee. In order to remedy this defect, other studies [13, 14, 17, 20, 21, 23] only analyzed the varus or valgus knee, but they did not consider the two types of under- and over-correction in the patients with non-neutral alignment after surgery. Three recent studies [18, 19, 24] have grouped preoperative varus osteoarthritis into neutral, mild varus, severe varus and valgus according to the postoperative alignment which made up for the above two shortcomings to a certain extent. However, this method can not analyzed the whole population. In addition, the clinical results about alignment on function are controversial.

Based on the above, we propose a new grouping method (under-correction, neutral and over-correction) which can reflects the preoperative knee condition and the change of coronal alignment. But there was no difference in the postoperative HSS score (88.27 vs 88.00 vs 85.62, *p* = 0.25)and the incremental scores (26.23 vs 25.22 vs 22.88, *p* = 0.54) among the three cohorts. Furthermore, the postoperative MFTA did not correlate with the clinical outcome score (R = − 0.10, *p* = 0.07).

In order to have a intuitive and clear index to predict knee function after TKR, on the basis of previous grouping method, we also innovatively introduced the concept of correction rate for the first time and tried to explore whether there is a correlation between correction rate and clinical outcome score for the severe preoperative vasus and valgus deformities. Mainstream opinions the relationship between coronal alignment and clinical outcomes for the patients with preoperative varus is that the postoperative residual mild varus alignment as well as neutral alignment led to excellent functional outcomes, but the postoperative severe varus and valgus alignment should be avoided [14, 18, 19, 24]. And some studies also reported that the restoration of a neutral alignment in preoperative severe deformities may be challenging and require more complex bone cuts and larger number of soft tissue releases [34], which in turn increased the injury of patients and lead to poor clinical outcomes. Therefore, according to the above conclusions, for preoperative severe varus deformities, we speculate that patients will get the best clinical outcomes when the correction rate is close to a certain value of 100%. This also mean the scatter plot of the clinical outcome score and the correction rate is likely to show the graphic distribution of the middle height and the gradual decrease of the two sides. But our analysis did not show any positive correlation between correction rate and HSS score as we expected (Fig. 6).

Based on the results of the current study, we believe that it is unrealistic to predict clinical outcome scores only according to coronal alignment. Only reaching the so-called neutral alignment after surgery may not mean a excellent clinical result. In our study, the patients who have not realized the neutral alignment also can get a good knee function, while not all the patients with neutral alignment got good clinical results. On the other hand, TKR is a soft tissue procedure and clinical outcome depends on many factors. As far as the characteristics of the patient are concerned, such as age, cardiopulmonary disease, other sequelae that keep the patient from walking very far at a time, and pain from the lumbar disease pain, the contralateral knee disease, the onset of rheumatoid arthritis, they also exert certain effects on the HSS score. Besides, the success of surgery is related to a variety of factors. In addition to the coronal alignment, the axial and rotational alignment, the balance and release of the ligaments, the fixation and rotation of the prosthesis, the rehabilitation of the patient, and varieties of environmental factors have played very important roles in TKR. It is difficult to standardize all these factors. Therefore, it is not likely to be of great clinical value to predict the postoperative knee function score according only to whether the alignment is normal, especially by just the concept of the 0° ± 3° safety zone.

Finally, with regard to BMI, we also found that patients with lower preoperative BMI are more likely to be corrected to a postoperative neutral alignment, perhaps further research is needed. Besides, for the preoperative MFTA, we found that the preoperative varus deformity of patients in under-correction group was more serious than that in the other two groups. This finding reflects the problem that even though some studies have reported that residual varus alignment does not compromise clinical outcomes or has better clinical outcomes after TKR, a neutral mechanical axis still should be the initial objective for a TKR. Because the anatomy of the varus knees often leads already to under-correction. This is consistent with some studies to some extent [35–37].

There are some limitations in our study. First, we only conducted a retrospective analysis of cases of a single center by one single surgeon in our institution, and there was a large difference in the sample size among the groups, especially in group C, which had fewer patients with over-correction, so the study might have some bias. Second, the patients were selected over a very long period (200722019) which gives an average of 20–30 cases/year. Implants and surgical techniques might have changed during this long time. However, there was no statistical difference in follow-up time among three cohorts and most patients underwent surgery after 2012. Third, some studies [38, 39] reported that the HSS score is an obsolete tool to evaluate TKR outcome with obvious risk of a ceiling effect, however our institution still use it to evaluate clinical outcomes and the results are almost the same as other scales such as Knee Society Scores (KSS). Fourth, we did not analyzed the impact of coronal alignment of tibial or femoral components on clinical outcome score due to insufficient data. Lastly, we did not comment on the impact of coronal alignment on prosthetic survivorship due to the mean follow-up of our study was 3.62 years. However, this is not the purpose of our study, and our aim is to assess the impact of alignment on clinical functional outcomes.

## Conclusion

TKR is a soft tissue procedure and clinical outcome depends on many factors, only reaching neutral alignment after surgery may not mean a good clinical result. Therefore, categorization of optimal coronal alignment after TKR may be impractical. But we still believe that the concept of correction rate and new grouping method are worthy of research which can reflects the preoperative knee condition and the change of coronal alignment. Perhaps it can be better used in TKR in the future.

## Data Availability

The datasets analysed during the current study are not publicly available because we will expand the sample size and extend the follow-up time to further explore the relationship between correction rate and clinical outcome, but these are available from the corresponding author on reasonable request.

## Abbreviations

TKR: Total knee replacement
HSS score: Hospital for Special Surgery score
MFTA: Mechanical femoral-tibial angle
KSS: Knee Society Scores
